# Influence of *KISS1* gene polymorphisms on the risk of polycystic ovary syndrome and its associated variables, in Saudi women

**DOI:** 10.1186/s12902-020-0537-2

**Published:** 2020-05-07

**Authors:** Maha H. Daghestani, Mazin H. Daghestani, Mamoon Daghistani, Khushboo Ambreen, Fadwa S. Albalawi, Lina M. AlNeghery, Arjumand S. Warsy

**Affiliations:** 1 Department of Zoology, Female Center for Scientific and Medical Colleges, King Saud University, Riyadh, Saudi Arabia; 2 Department of Obstetrics & Gynaecology, Umm-Al-Qura University, Makkah, Saudi Arabia; 3 Department of Surgery, King Abdulaziz Medical City, National Guard Heath Affairs, Jeddah, Saudi Arabia; 4 Department of Biotechnology, Integral University, Lucknow, India; 5 Department of Biology, College of Science, Imam Mohammad Ibn Saud Islamic University, Riyadh, Saudi Arabia; 6 Central Laboratory, Female Center for Scientific and Medical Colleges, King Saud University, Riyadh, Saudi Arabia

**Keywords:** Polycystic ovary syndrome, *KISS1* gene polymorphisms, Kisspeptin, LH, FSH, Waist-hip ratio

## Abstract

**Background:**

Polycystic ovary syndrome (PCOS) is a complex multifactorial disorder, affecting millions of women worldwide. The role of genetic polymorphisms of *the KISS1* gene on the development of PCOS is still obscure. This study was designed to investigate the probable influence of *KISS1* gene polymorphisms on PCOS and its associated variables: BMI, waist-hip ratio, kisspeptin, LH, FSH, and LH-FSH ratio.

**Methods:**

The study comprised 104 PCOS women and 109 controls, with age ranging from 19 to 36 years. BMI, waist-hip ratio, and circulating levels of kisspeptin, LH, and FSH were measured. DNA was extracted, and genotyping of *the KISS1* gene was carried out by nucleotide sequencing. The PCOS-associated variables were analyzed in different genotypes of single nucleotide polymorphisms (SNPs) of *the KISS1* gene.

**Results:**

The values of waist-hip ratio (WHR), LH, and LH-FSH ratio were significantly higher in PCOS women than controls. BMI, kisspeptin, and FSH levels exhibited no significant difference between the groups. Six novel SNPs of *KISS1* gene were identified. Three: rs372790354G > A, rs12998G > A, and rs35431622A > T were investigated. Among these SNPs, the genotype and allele frequencies of rs372790354 showed significant association with PCOS (GA: *p* = 0.018, AA: *p* = 0.022, mutant allele-A: *p* = 0.021) and the G allele was protective. The values of LH, kisspeptin, and WHR of PCOS women were significantly influenced (*p* < 0.05) by the AA genotype of rs372790354. The other two SNPs rs12998G > A and rs35431622A > T revealed no significant influence on PCOS and associated variables. Haplotypes were constructed, but there was no significant difference between the patients and controls.

**Conclusion:**

In conclusion, this is the first study, which reports a significant influence of *KISS1* gene polymorphism (rs372790354G > A) on PCOS and its associated variables. However, more extensive research is necessary to confirm these findings.

## Background

Polycystic ovary syndrome (PCOS) is one of the notable common health concerns in the female society of reproductive age ranging from 12 to 45 years with a highly variable prevalence rate of 2.2 to 26% in different ethnic groups [1]. It is a complex endocrine disorder characterized by chronic anovulation, hyperandrogenism, and polycystic ovarian morphology and associated primary clinical manifestations of irregular or missed periods, and excessive hair growth on the face and body [2]. In addition to these endocrinal factors and its related health complications, the complex etiology of PCOS is also influenced by the involvement of genetic, metabolic, and environmental factors [3].

In recent years, considerable research efforts have been made towards understanding the multifactorial etiology of PCOS. In this regard, genetic understanding of this disease in terms of hypothalamic-pituitary-gonadal (HPG) axis associated genetic factors, has received considerable attention by the researchers [4].

Many previous studies have explored mutations in the HPG axis linked various genes such as *KISS1*, *GPR54* receptor, *GnRHR*, *LHR* and *FSHR*, for the development of PCOS in the adult female population [5–7]. Among these genes, *KISS1* has emerged as one of the candidate genes contributing to a regulatory role in the female reproductive system with an essential function in gonadotropin secretion of the HPG axis [8]. Some single nucleotide polymorphisms (SNPs) in the *KISS1* gene are found to disrupt the healthy functioning of the female reproductive system through disturbing the HPG axis and are postulated to play an essential role in PCOS etiopathogenesis [5, 9].

The effects of *KISSI* gene polymorphisms on susceptibility to PCOS have been investigated in two previous studies [9, 10]. However, until now, no adequately powered study is available in the literature, which confidently reveals the role of any relevant polymorphism of *the KISS1* gene in PCOS pathogenesis.

Biochemically, ovarian dysfunction of PCOS is reflected in the excessive production of luteinizing hormone (LH) and a normal or low level of follicle-stimulating hormone (FSH) from the anterior pituitary [11]. Kisspeptins are neuropeptides of different amino acid lengths (Kp-54, Kp-14, Kp-13, and Kp-10), encoded by the *KISS1* gene, and unveils a vital regulatory role in the function of the HPG-axis, by utilizing G protein-coupled receptor known as GPR54 [12]. The major pathway of kisspeptin is the direct action on GnRH neurons to release GnRH into the portal circulation, which in turn induces the stimulatory activity of LH and FSH with the release of androgens [13]. Hence, the evaluation of the circulating levels of kisspeptin, LH, and FSH in susceptible women may be of importance in understanding the multifactorial etiology of PCOS.

Likewise, elevated values of body mass index (BMI) and waist-hip ratio (WHR) are considered as conventional anthropometric markers for the feature of obesity. They are proved to be linked with PCOS risk by directly creating disturbances in metabolic as well as endocrine system [14, 15]. Many previous reports also emphasize the significance of BMI and waist-hip ratio as the markers of obesity, having responsibility for the co-morbidity events of PCOS [16–18].

Hence, *KISS1* gene polymorphisms, kisspeptin, LH, FSH, BMI, and waist-hip ratio are the essential interlinking factors that exert their influence on the expression of PCOS. The current study investigates the values of BMI, waist-hip ratio, and the blood levels of kisspeptin, LH, FSH, and the ratio of LH-FSH, in PCOS patients and controls. In an attempt to determine the influence of the *KISS1* gene polymorphisms on the development of PCOS, the study assesses the genotype and allele frequencies of SNPs of the *KISS1* gene for both study groups. Furthermore, we have attempted to demonstrate the possible influence of the *KISS1* gene polymorphisms on PCOS associated endocrine and obesity-linked variables (kisspeptin, LH, FSH, LH-FSH ratio BMI, and waist-hip ratio).

## Methods

### Study subjects

The subjects of this present case-control study consisted of 104 women with well-characterized manifestations of PCOS and 109 control healthy women having no clinical history of PCOS. The study was approved by the Ethical Committee of the Institutional Review Board (IRB), Umm Al-Qura University, Makkah, Saudi Arabia (IRB No. 235). Study participants with an age range of 19–36 years, were recruited from different private hospitals of Makkah, Kingdom of Saudi Arabia.

Enrolled PCOS patients were newly diagnosed, and the criteria for the diagnosis of the PCOS patients was based on two of the three following features, according to Rotterdam 2003 criteria: (I) oligomenorrhea (menstrual period length greater than 35 days) or amenorrhea (menstrual period absent for six months), (II) clinical and/or biochemical signs of hyperandrogenism, (III) polycystic ovaries morphology as seen on ultrasound (at least one ovary contained > 12 follicles measuring 2–9 mm in diameter and/or increased ovarian volume of at least 10 ml) [19].

Patients having other reasons, of hyperandrogenism or menstrual irregularity such as Cushing’s syndrome, prolactinoma, and congenital adrenal hyperplasia were excluded. Pregnancy cases and women with the first postpartum year were also not included in our study. Patients having received any kind of hormonal medicine (such as oral contraceptives, ovulation-inducing agents, and anti-androgens) in the previous 3 months of the research, were excluded.

The selection of the control subjects was based on the enrollment of the healthy women population having no history of PCOS with the regular menstrual cycle (menstrual period up to 7 days and menstrual cycle of 21–35 days), normal androgen levels and no symptoms of hirsutism. The control women were matched with PCOS women for age and BMI.

To collect demographic and clinical data of the patients, we prepared a specially designed questionnaire, and the demographic characteristics (age, weight, height, waist, and hip measurements), history of the menstrual cycle, hirsutism, acne, and previous disease and drug history were recorded. For patients with doubtful records, biochemical laboratory tests and ultrasound investigations were performed. The confirmed cases of PCOS were recruited. Controls were screened for biochemical signs of raised androgens, ultrasonic ovarian morphology, and menstrual health records. Only those with normal results were included in the study.

For each enrolled woman, BMI (weight in kg divided by height in m^2^) was calculated. Likewise, the calculation of waist-hip ratio was based on measurements of waist circumference by an experienced nurse (the narrowest circumference between the lower costal margins and the iliac crest) and hip circumference (the maximum circumference at the level of the femoral trochanters) with the proper standing position of the study subjects.

### Sample collection

In both groups of the study subjects, the blood samples were collected between days 3 and 6 of the menstrual cycle, at about 8:00 am. Each subject was requested to attend the clinic in a fasting state, and 10 ml of the blood sample was drawn from each subject using the venipuncture method. A total of 5 ml of this blood was transferred in an EDTA coated vial, and immediately processed for obtaining buffy coat and plasma, through centrifugation process. Buffy coat was used for DNA extraction, and the plasma was used for the estimation of kisspeptin. Remaining 5 ml of the blood sample was drawn in a red top tube and used to obtain serum by centrifugation process, for the evaluation of FSH and LH. All samples were consequently stored at -80oC until experimental analysis.

### Estimation of kisspeptin, FSH and LH levels

The blood plasma level of kisspeptin was measured using an enzyme-linked immunosorbent assay (ELISA) kit from Phoenix Pharmaceuticals Inc., Belmond, CA, following extraction with Phoenix Peptide sep-columns (RK-Sepcol-2). Blood serum levels of FSH and LH were also measured by a specific ELISA kit (Human, Cat. No.65205.GER).

### *KISS1* genotyping analysis

**Fig. 1:**
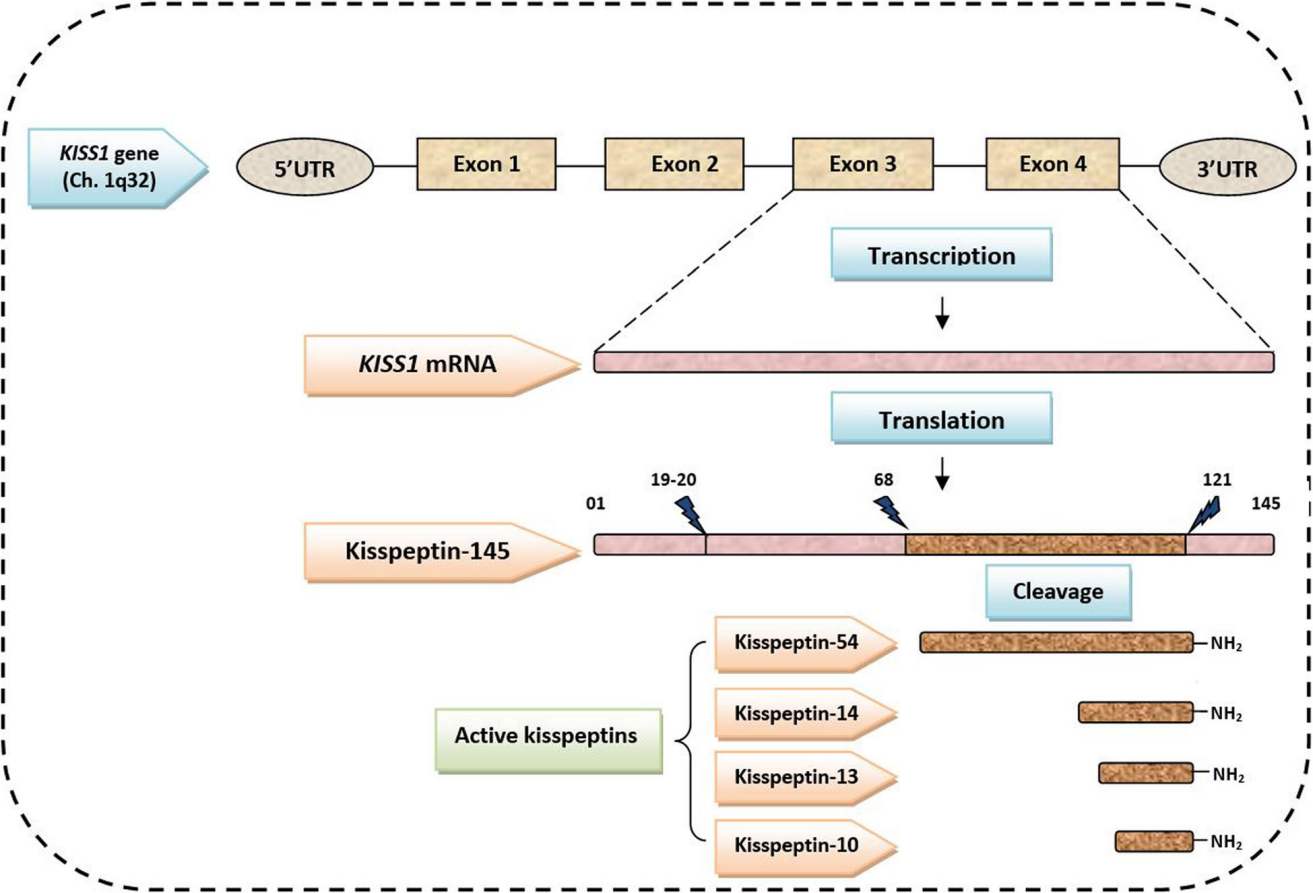
Representation of the Human *KISS1* gene with its exons and protein product (kisspeptin). THE Human *KISS1* gene is mapped on chromosome 1q32. It consists of four exons, of which only third and fourth exons are finally translated into the 145 amino acid peptide, and it is cleaved into four forms of active kisspeptin containing 54, 14, 13, and 10 amino acids

*KISS1* gene is mapped on chromosome 1q32 with its four exons (Fig. 1). Genomic DNA from 104 PCOS patients and 109 controls were obtained, and PCR sequencing was performed for obtaining SNPs of *the KISS1* gene. For this purpose, the buffy coat was used to extract the genomic DNA, utilizing a DNA extraction kit from Puregene (Puregene Blood Kit, QIAGEN, Cat. No. 158389, USA). Polymerase chain reaction (PCR) was performed using the following forward and reverse primers: F: 5′-ACCTGCCGAACTACA ACTGG-3, R: 5′-TGAAGGAACAGGCGGTTAGT-3′, under the following conditions: initial denaturation step at 95 °C for 15 min, followed by 34 cycles of denaturation at 95 °C for 1 min, annealing at 60 °C for 1 min, and extension at 72 °C for 1 min, with the final extension of 10 min at 72 °C. The size of the PCR product was 353 bp. The nucleotide sequencing of the PCR product was carried out using the ABI Big Dye Terminator protocol on ABI 3100 Avant Genetic Analyzer.

### Statistical analysis

The statistical analyses were conducted using SPSS for Windows (version 20.0). The descriptive characteristics of the different continuous variables were expressed as mean ± SD. The Student’s t-test was used to obtain the significance of the difference in the mean values of continuous variables between PCOS patients and controls. Genotype and allele frequencies of the *KISS1* gene polymorphisms were calculated for both study groups, and the significance of the difference was determined using the Chi-Square test. Odds ratio (OR), its 95% confidence interval (CI), and *p*-values were obtained. Hardy Weinberg equilibrium was tested.

The levels of PCOS associated variables were analyzed in the different genotypes of each identified SNP of the *KISS1* gene. The Pairwise t-test was used to assess the significance of the difference between wild-type homozygous and mutant allele carriers (mutant homozygous and heterozygous). A *p*-value < 0.05 was considered as statistically significant.

## Results

### Demographic and endocrine characteristics of study subjects

The demographic characteristics of PCOS patients and controls are presented in Table 1. A significant difference was noted between PCOS patients and controls, only in the value of the waist-hip ratio (*p* < 0.001) while the age (*p* = 0.88) and BMI (*p* = 0.63) exhibited no significant difference between the study groups.

**Table 1:** Demographic and endocrine characteristics of study subjects

Characteristics	PCOS Patients (***n*** = 104) (Mean ± SD)	Controls (***n*** = 109) (Mean ± SD)	***p***-value
**Age (Years)**	25.06 ± 3.88	24.96 ± 5.56	0.886
**Waist (cm)**	87.86 ± 13.82	86.15 ± 17.09	0.422
**Hip (cm)**	104.39 ± 11.39	110.48 ± 14.78	0.001*
**Waist-Hip ratio**	0.84 ± 0.07	0.77 ± 0.06	0.0001*
**BMI (Kg/m**^**2**^**)**	29.45 ± 5.99	29.89 ± 7.31	0.633
**Kisspeptin (ng/ml)**	0.41 ± 0.16	0.39 ± 0.07	0.415
**FSH (IU/L)**	5.09 ± 1.54	4.83 ± 1.55	0.223
**LH (IU/L)**	14.43 ± 7.48	4.56 ± 1.38	0.0001*
**LH-FSH ratio**	2.94 ± 1.07	0.98 ± 0.37	0.0001*

*Significant, *p* values refer to Student’s t-test; *LH* luteinizing hormone, *FSH* follicle stimulating hormone, *BMI* body mass index

As shown in Table 1, the levels of kisspeptin (*p* = 0.415) and FSH showed (*p* = 0.223) no significant difference between PCOS patients and controls. A significant elevation in the level of LH for the PCOS patients (*p* = 0.0001) was the most noticeable result, as the level was more than three-fold higher in PCOS patients than in the controls. Furthermore, when both groups were examined for the values of the LH-FSH ratio, a significant difference (*p* = 0.0001) was noted.

### *KISS1* gene polymorphisms

A total of six SNPs were identified through sequencing of the *KISS1* gene. The results of three SNPs (rs372790354 G/A, rs12998 G/A, and rs35431622 A/T) (Table 2), are presented in this paper. The SNP rs372790354 G/A was novel and was located in the 5′- untranslated region (5‘UTR). The other two SNPs, rs12998 G/A, and rs35431622 A/T were also characterized as novel SNPs, and these were positioned in the exon 1 and exon 2, respectively. The rs12998 G/A is a non-synonymous SNP and results in the substitution of Glutamic acid at position 20 by a basic amino acid Lysine. The third identified SNP rs35431622 A/T was also a non-synonymous which results in the substitution of Glutamine with Leucine at position 36.

**Table 2:** Single nucleotide polymorphisms identified in the *KISS1* gene by sequencing

db SNP ID	Chromosome Position	mRNA position	Heterozygosity	MAF	Allele	Location	Function	Amino-acid change
rs372790354	Chr1:204196427	–	–	–	G > A	5 Prime UTR variant	NA	–
rs12998	Chr1:204192819	212	0.093	0.023	G > A	Exon 1	Non-synonymous	p.Glu20Lys
rs35431622	Chr1:204190794	261	0.103	0.075	A > T	Exon 2	Non-synonymous	p.Gln36Leu

*db SNP ID* single nucleotide polymorphism Database ID, *MAF* minor allele frequency

### Genotype and allele frequencies of the *KISS1* gene polymorphisms

The genotype and allele frequencies of the identified SNPs of *the KISS1* gene are presented in Tables 3 and 4. The frequencies of the genotypes (GA and AA) of rs372790354 were significantly different (*p* < 0.05) between PCOS patients and controls. The GA genotype occurred at a lower frequency among PCOS patients as compared to controls, while the genotype AA was observed only in the PCOS patients. The G allele of rs372790354 was protective, and the frequency of allele A was significantly (*p* = 0.021) higher in PCOS patients compared to controls (Table 4). The Hardy-Weinberg equilibrium was maintained for PCOS and control subjects with *p*-values of > 0.05 and 1. 00, respectively.

**Table 3:** Genotype frequencies of studied SNPs of the *KISS1* gene, in PCOS patients and controls

SNPs	Genotypes	Frequency			OR (95%CI)	χ^**2**^	***p***-value
		PCOS patients n (%)	Controls n (%)	Total n (%)			
**rs372790354 G/A**	GG	86 (82.7%)	92 (84.4%)	178 (83.6%)	Reference		
	GA	13 (12.5%)	17 (15.6%)	30 (14.1%)	0.07 (0.01–1.38)	5.51	0.018*
	AA	5 (4.8%)	0 (0.0%)	5 (2.3%)	1.17 (0.64–215)	5.20	0.022*
	**Total**	104 (100.0%)	109 (100.0%)	213 (100.0%)			
**rs12998 G/A**	GG	87 (83.7%)	88 (80.7%)	175 (82.2%)	Reference		
	GA	12 (11.5%)	17 (15.6%)	29 (13.6%)	0.56 (0.13–2.55)	0.56	0.454
	AA	5 (4.8%)	4 (3.7%)	9 (4.2%)	1.26 (0.33–4.86)	0.12	0.732
	Total	104 (100.0%)	109 (100.0%)	213 (100.0%)			
**rs35431622 A/T**	AA	97 (93.3%)	101 (92.7%)	198 (92.96%)	Reference		
	AT	7 (6.7%)	8 (7.3%)	15 (7.04%)	0.91 (0.32–2.61)	0.03	0.862
	TT	0 (0.0%)	0 (0.0%)	0 (0.0%)	–	–	–
	Total	104 (100.0%)	109 (100.0%)	213 (100.0%)			

*Significant*, p* values refer to Chi-Square (**χ**^**2**^) test, *OR* odds ratio, *CI* confidence interval

**Table 4:** Allele frequencies of studied SNPs of the *KISS1* gene, in PCOS patients and controls

SNPs	Alleles	Frequency			OR (95%CI)	χ^**2**^	***p***-value
		PCOS patients (%)	Controls (%)	Total (%)			
**rs372790354 G/A**	G	88.9%	92.2%	90.6%	0.680 (0.35–1.31)	1.33	0.021*
	A	11.1%	7.8%	9.4%			
	**Total**	100.0%	100.0%	100.0%			
**rs12998 G/A**	G	89.4%	88.5%	88.97%	1.09 (0.59–2.01)	0.09	0.769
	A	10.6%	11.5%	11.03%			
	**Total**	100.0%	100.0%	100.0%			
**rs35431622 A/T**	A	96.6%	96.3%)	96.5%	1.09 (0.38–3.07)	0.03	0.864
	T	3.4%	3.7%	3.5%			
	**Total**	100.0%	100.0%	100.0%			

*Significant*, p* values refer to Chi-Square (**χ**^**2**^) test, *OR* odds ratio, *CI* confidence interval

For the SNP rs12998, the genotype frequencies were not significantly (*p* > 0.05) different between PCOS patients and controls. The GA genotype was more frequently observed in controls compared to PCOS patients, but the difference was not statistically significant. This SNP exhibited the Hardy-Weinberg equilibrium (*p* > 0.05) for both study groups.

Likewise, the genotypes TA and AA of the polymorphism rs35431622 showed no significant influence on the risk of PCOS. The homozygous genotype TT was not identified in the PCOS patients and controls. The Hardy-Weinberg equilibrium was maintained (*p* > 0.05) for this SNP in both the PCOS and control subjects. As illustrated in Table 4. the allele frequencies of these two SNPs (rs12998 G/A and rs35431622 A/T) were not significantly different between PCOS patients and controls.

### Influence of *KISS1* gene polymorphisms on PCOS associated endocrine and obesity linked variables

To study the probable impact of the three studied SNPs (rs372790354 G/A, rs12998 G/A, and rs35431622 A/T) of *the KISS1* gene, on the levels of PCOS associated variables, the PCOS patients and controls were grouped based on their genotypes. The values of endocrine (LH, FSH, LH-FSH ratio, and kisspeptin) and obesity-linked parameters (BMI and waist-hip ratio), were determined in the different genotypes. The comparison analysis was performed between wild type homozygous and mutant allele carriers (mutant homozygous and heterozygous). The results are presented in Tables 5 and 6. The SNP rs372790354 was revealed to exhibit a significant influence (*P* < 0.05) on the endocrine (LH and kisspeptin) and obesity-linked (waist-hip ratio) parameters of PCOS patients. While other parameters (FSH, LH-FSH ratio, and BMI) of PCOS patients were not influenced by rs372790354. When the influence of this SNP on the studied parameters of control subjects was investigated, no significant difference was obtained (Table 5).

**Table 5:** Influence of rs372790354 polymorphism of *KISS1* gene on Endocrine and Obesity linked parameters, in PCOS patients and controls

Parameters		Genotypes of rs372790354			***P***-value (Pair wise Comparison)
		GG	GA	AA	
	No. C	***n*** = 92	***n*** = 17	***n*** = 0	
	No. P	***n*** = 86	***n*** = 13	***n*** = 5	
**Obesity linked Parameters**					
BMI (Kg\m^2^)	C	29.88 ± 7.21	29.89 ± 8.06	–	GG/GA = 0.776
					–
	P	29.43 ± 5.90	29.62 ± 5.01	29.40 ± 10.4	GG/GA = 0.902
					GG/AA = 0.991
Waist-hip ratio	C	0.77 ± 0.06	0.79 ± 0.07	–	GG/GA = 0.389
					–
	P	0.83 ± 0.07	0.84 ± 0.08	0.88 ± 0.16	GG/GA = 0.925
					GG/AA< 0.05*
**Endocrine Parameters**					
FSH (IU\L)	C	4.76 ± 1.60	5.17 ± 1.22	–	GG/GA = 0.063
					–
	P	5.16 ± 1.53	4.41 ± 1.08	5.52 ± 2.39	GG/GA = 0.092
					GG/AA = 0.624
LH (IU\L)	C	4.53 ± 1.45	4.68 ± 1.02	–	GG/GA = 0.135
					–
	P	12.54 ± 2.55	11.85 ± 3.43	14.93 ± 8.02	GG/GA = 0.051
					GG/AA< 0.05*
LH-FSH ratio	C	0.99 ± 0.39	0.94 ± 0.26	–	GG/GA = 0.277
					–
	P	2.96 ± 1.08	2.91 ± 1.12	2.45 ± 0.62	GG/GA = 0.881
					GG/AA = 0.144
Kisspeptin (ng\ml)	C	0.42 ± 0.15	0.37 ± 0.06	–	GG/GA = 0.106
					–
	P	0.41 ± 0.16	0.42 ± 0.15	0.28 ± 0.19	GG/GA = 0.887
					GG/AA< 0.05*

*Significant, *C* control, *P* PCOS patients, *BMI* Body mass index, *LH* luteinizing hormone, *FSH* follicle stimulating hormone, *p* values indicate comparison of features between wild-type homozygous and mutant allele carriers (mutant homozygous and heterozygous)

**Table 6:** Influence of rs12998 and rs35431622 polymorphisms of *KISS1* gene on Endocrine and Obesity linked parameters, in PCOS patients and controls

Parameters		Genotypes of rs12998			***P***-value (Pair wise Comparison)	Genotypes of rs35431622		***P***-value (Pair wise Comparison)
		GG	GA	AA		AA	AT	
	No. C	***n*** = 88	***n*** = 17	***n*** = 4		***n*** = 101	***n*** = 8	
	No. P	***n*** = 87	***n*** = 12	***n*** = 5		***n*** = 97	***n*** = 7	
**Obesity linked Parameters**								
BMI (Kg\m^2^)	C	29.82 ± 7.04	30.45 ± 9.33	29.00 ± 4.02	GG/GA = 0.752	29.54 ± 7.18	34.27 ± 7.95	AA/AT = 0.987
					GG/AA = 0.503			
	P	29.44 ± 5.86	28.88 ± 5.48	31.04 ± 9.96	GG/GA = 0.748	29.55 ± 6.05	28.11 ± 5.32	AA/AT = 0.987
					GG/AA = 0.740			
Waist-hip ratio	C	0.77 ± 0.07	0.79 ± 0.07	0.79 ± 0.06	GG/GA = 0.218	0.77 ± 0.06	0.79 ± 0.09	AA/AT = 0.640
					GG/AA = 0.420			
	P	0.84 ± 0.07	0.81 ± 0.07	0.89 ± 0.15	GG/GA = 0.254	0.84 ± 0.07	0.81 ± 0.06	AA/AT = 0.965
					GG/AA = 0.317			
**Endocrine Parameters**								
FSH (IU\L)	C	4.73 ± 1.54	5.04 ± 1.48	6.10 ± 1.83	GG/GA = 0.434	4.84 ± 1.50	4.61 ± 2.16	AA/AT = 0.070
					GG/AA = 0.230			
	P	5.11 ± 1.53	4.59 ± 1.26	5.80 ± 2.18	GG/GA = 0.212	5.07 ± 1.53	5.20 ± 1.77	AA/AT = 0.972
					GG/AA = 0.343			
LH (IU\L)	C	4.55 ± 1.42	4.43 ± 1.25	5.20 ± 1.53	GG/GA = 0.738	4.54 ± 1.35	4.81 ± 1.86	AA/AT = 0.163
					GG/AA = 0.460			
	P	14.88 ± 8.03	11.36 ± 2.47	13.82 ± 2.67	GG/GA = 0.135	14.46 ± 7.69	13.95 ± 3.72	AA/AT = 0.466
					GG/AA = 0.488			
LH-FSH ratio	C	0.99 ± 0.38	0.94 ± 0.38	0.87 ± 0.25	GG/GA = 0.526	0.97 ± 0.37	1.11 ± 0.34	AA/AT = 0.798
					GG/AA = 0.534			
	P	2.97 ± 1.06	2.82 ± 1.29	2.55 ± 0.71	GG/GA = 0.650	2.94 ± 1.09	2.85 ± 0.88	AA/AT = 0.695
					GG/AA = 0.263			
Kisspeptin (ng\ml)	C	0.39 ± 0.07	0.37 ± 0.05	0.40 ± 0.10	GG/GA = 0.150	0.39 ± 0.06	0.41 ± 0.07	AA/AT = 0.811
					GG/AA = 0.946			
	P	0.42 ± 0.16	0.38 ± 0.14	0.31 ± 0.25	GG/GA = 0.399	0.41 ± 0.16	0.35 ± 0.11	AA/AT = 0.184
					GG/AA = 0.156			

*Significant, *C* control, *P* PCOS patients, *BMI* Body mass index, *LH* luteinizing hormone, *FSH* follicle stimulating hormone, *p* values indicate comparison of features between wild-type homozygous and mutant allele carriers (mutant homozygous and heterozygous). For SNP rs35431622: TT = 0, in both PCOS patients and controls

The other two SNPs: rs12998 G/A, and rs35431622 A/T, exhibited no significant influence on the values of endocrine as well as obesity liked parameters in the PCOS patients. Likewise, in the control group, no significant differences were observed for the levels of the studied parameters between the genotypes of these two SNPs (Table 6).

Haplotype analysis was carried out using SNPstat, and the results are presented in Table 7. Five haplotypes exhibited polymorphism in the PCOS and control group. The most frequently identified haplotype in both groups was GGA (rs12998, rs372790354, and rs35431622), but the results in the two groups were not significantly different. None of the other haplotypes showed any significant difference between the patients and controls.

**Table 7:** Construction of haplotypes of the three studied SNP in the PCOS patients and control group

Serial No.	Haplotype			Freq.	Freq.	OR (95% CI)	*P*-value
	SNP						
	rs12998	rs372790354	rs35431622	Control	Case		
1	G	G	A	0.8413	0.8302	1.00	–
2	A	A	A	0.0508	0.0665	1.22 (0.56–2.63)	0.62
3	A	G	A	0.0516	0.0393	0.82 (0.37–1.78)	0.61
4	G	A	A	0.0196	0.0304	1.43 (0.47–4.33)	0.53
5	G	G	G	0.0244	0.02	0.74 (0.18–3.05)	0.68

## Discussion

The present study describes the probable influence of three identified SNPs (rs372790354 G/A, rs12998 G/A, and rs35431622 A/T) in *the KISS1* gene in PCOS pathogenesis. Importantly, for the first time, we have attempted to demonstrate the influence of these three novel SNPs on the endocrine (kisspeptin, LH, FSH, LH-FSH ratio) and obesity-linked parameters (BMI and waist-hip ratio), in women with and without PCOS.

The genotype and allele frequencies were calculated and compared between PCOS patients and controls to elucidate the role of these studied SNPs in PCOS. For the SNP rs372790354, the genotypes AA and GA exhibited significant association with the risk of PCOS. The mutant AA genotype was found only in PCOS patients, and the frequency of the GA genotype was significantly higher in control subjects. The allele G showed a protective effect.

In recent years, *the KISS1* gene has been addressed as one of the crucial regulators in controlling the function and maturation of the reproductive system [20]. This gene consists of four exons, of which only third and fourth exons are finally translated into 145 amino acids and is cleaved into shorter four forms of kisspeptin containing amino acids of 54, 14, 13, and 11 and exhibit the same affinity for their receptor-GPR54 [8]. The role of kisspeptin (a major product of *KISS1* gene) has been well identified in puberty, ovulation, brain sex differentiation, and fertility, with an essential regulatory function in the normal release of hypothalamic GnRH and consequently in LH secretion [21, 22]. The pathophysiological mechanism of PCOS is also reflected in the inappropriate GnRH/LH secretion [23, 24]. However, clear evidence for the role of *KISS1*/kisspeptin expression in the mechanism of PCOS pathogenesis is still not obtained.

In this study, the results showed that the SNP rs372790354, which is significantly different in PCOS patients, is located in the 5’UTR (untranslated region) of *the KISS1* gene. The 5’UTR has been recognized as playing an important role in the regulation of gene expression through regulating the process of RNA transcription, mRNA stability and its localization, and translational efficiency [25, 26]. Moreover, 5’UTR efforts great contribution in the initiation step of translation. The multi-step process of gene expression is primarily depended on this initiation translation step [27].

Keeping in view the role of 5’UTR in gene expression regulation, our study has hypothesized that the rs372790354 polymorphism of *the KISS1* gene, can dysregulate the multi-step process of gene expression. This step may indirectly promote alteration in the functional activity of *the KISS1* gene product- kisspeptin and its receptor GPR54. The clear evidence for the considerable influence of the SNP rs372790354 on kisspeptin level of PCOS women is illustrated in Table 4, which highlights a significant difference in kisspeptin level, between homozygous wild-type (GG) and mutant (AA) genotypes of this SNP. While in the control group, the level of kisspeptin was not influenced by any of the genotypes of rs372790354.

Moreover, when both study groups were compared for the level of kisspeptin (Table 1), no significant difference was obtained between PCOS patients and controls. No significant elevation of the kisspeptin level in PCOS patients in our study may be due to the small sample size. One of our previous studies, reported by Albalawi et al. [9], also highlights no significant difference in the level of kisspeptin between PCOS women and controls. It showed that the SNP rs4889 exhibited a significant influence on the kisspeptin level. Several previous studies have also shown a non-significant difference in the level of kisspeptin in women with PCOS as compared to non-PCOS women [28, 29]. Thus, these observations of our study suggest that the kisspeptin level of PCOS women was not significantly higher as compared to controls, but is significantly influenced by the SNP rs372790354 of *KISS1* gene.

It is well established that PCOS has a complex multifactorial etiology and is associated with increased secretion of LH, normal or low level of FSH, and an increased ratio of LH-FSH [30, 31]. In our study, PCOS patients showed significantly higher values of LH and LH-FSH ratio as compared to controls. While the level of FSH was almost the same between the two study groups. Likewise, many previous reports highlighted a significantly elevated level of LH and increased LH-FSH ratio in PCOS subjects with no significant alteration in the FSH level, compared to controls [32, 33]. Importantly, when the influence of the SNP rs372790354 on endocrine parameters (LH, FSH, and LH-FSH ratio) was investigated, it was seen that the level of LH in the PCOS group was influenced by this SNP and was significantly higher in mutant AA genotype. While, in the control group, no significant influence of SNP rs372790354 was observed. Likewise, the level of FSH and LH-FSH ratio showed no significant difference in the genotypes of this SNP in both study groups. These results suggest a significant influence of the AA genotype of rs372790354 on increased LH levels of PCOS women.

Results of several previous studies show that the pathology of PCOS is influenced by the obesity-linked parameters, including BMI and WHR [34, 35]. Hence, we also determined the values of BMI and WHR in both PCOS and control subjects and then observed any possible effects of rs372790354 on these obesity-linked parameters. Our results showed that there was no difference in BMI in the two groups, but WHR was significantly different. These findings confirm the results of other recent studies [17, 36]. Further, when the influence of rs372790354 was investigated on BMI and WHR, only WHR in PCOS patients showed a significant difference between the genotypes (GG and AA).

Based on these findings, we suggest that the rs372790354 polymorphism of *the KISS1* gene may have a direct effect on the functional activity of kisspeptin in terms of its altered behavior towards receptor GPR54. Consequently, the disturbed kisspeptin-GPR54 pathway encourages dysregulation in GnRH secretion, which further leads to the hypersecretion of LH. Subsequently, the condition of hyperandrogenism with a high level of testosterone is induced by the direct action of high stimulation of LH on gonads. Eventually, all these conditions may favor the increase in the risk of PCOS.

Many recent reports have suggested the role of altered behavior of kisspeptin as a major cause in inducing hyperandrogenism associated with PCOS [3, 37]. In this present study, we have suggested the role of SNP rs372790354 of *the KISS1* gene in creating a disturbance in the functional activity of kisspeptin with hypersecretion of LH, which in turn may be responsible for increasing the risk of PCOS. No other previous studies are available in the literature to compare the results of our study. However, the association of this polymorphism with PCOS should be confirmed by further studies, as the sample size in this study is relatively small and does not give strong evidence for these related findings.

The other identified SNPs rs12998 G/A, and rs35431622 A/T showed no significant association with the risk of PCOS. They did not exhibit any influence on the endocrine and obesity-linked parameters in PCOS patients and controls.

## Conclusion

In conclusion, the current study reports three novel SNPs (rs372790354 G/A, rs12998 G/A, and rs35431622 A/T) in the *KISS1* gene, among women population of Saudi Arabia and shows a significant influence of rs372790354 G/A on the risk of PCOS. Furthermore, this novel SNP shows a considerable impact on the levels of LH, kisspeptin, and WHR in PCOS women. These findings may improve the understanding of PCOS in regards to its multifactorial etiology, especially those associated with *KISS1* gene polymorphisms and their influence on the levels of biochemical and demographic factors. This study has the drawback that it was conducted on only a small number of samples. Hence, further, more extensive studies in different populations are required to confirm this association.

## Data Availability

The datasets used and/or analysed during the current study are available from the corresponding author on reasonable request.

## Abbreviations

BMI: Body mass index
LH: Luteinizing hormone
FSH: Follicle stimulating hormone
UTR: Untranslated region
MAF: Minor allele frequency
SNP: Single nucleotide polymorphisms
